# Solid state NMR studies of gels derived from low molecular mass gelators

**DOI:** 10.1039/c6sm00969g

**Published:** 2016-06-17

**Authors:** Nonappa, E. Kolehmainen

**Affiliations:** a Department of Applied Physics , Aalto University School of Science , Puumiehenkuja 2 , Espoo , FI-02150 , Finland . Email: nonappa@aalto.fi; b Department of Chemistry , University of Jyväskylä , Jyväskylä , FI-41004 , Finland . Email: erkki.t.kolehmainen@jyu.fi

## Abstract

Since its invention more than six decades ago, nuclear magnetic resonance (NMR) spectroscopy has evolved as an inevitable part of chemical as well as structural analysis of small molecules, polymers, biomaterials and hybrid materials. In the solution state, due to the increased viscosity of complex viscoelastic fluids such as gels, liquid crystals and other soft materials, the rate of molecular tumbling is reduced, which in turn affects the chemical shift anisotropy, dipolar and quadrupolar interactions. As a consequence the solution state NMR spectra show broad lines, and therefore, extracting detailed structural information is a challenging task. In this context, solid state (SS) NMR has the ability to distinguish between a minute amount of polymorphic forms, conformational changes, and the number of non-equivalent molecules in an asymmetric unit of a crystal lattice, and to provide both qualitative as well as quantitative analytical data with a short-range order. Therefore, SS NMR has continued to evolve as an indispensable tool for structural analysis and gave birth to a new field called NMR crystallography. Solid state cross polarization (CP) and high resolution (HR) magic angle spinning (MAS) NMR spectroscopy has been used to study weak interactions in polymer gels. However, the application of SS NMR spectroscopy to study gels derived from low molecular weight gelators has been limited until recently. In this review, we will focus on the importance of solid state NMR spectroscopy in understanding and elucidating the structure of supramolecular gels derived from low molecular weight gelators with selected examples.

## Abstract 2

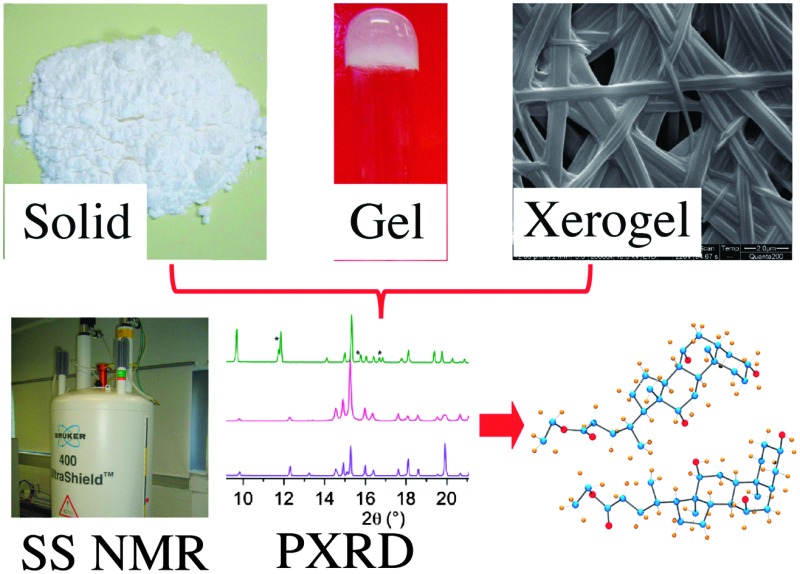
The emergence of NMR crystallography provides a unique opportunity to study solids, gels and xerogels, thereby providing ample information to elucidate molecular packing in the native gel. This review details the importance as well as the application of solid state NMR spectroscopy combined with other analytical tools to study gels derived from low molecular mass organo- and hydrogelators.

## Introduction

Weak non-covalent interactions are ubiquitous in nature and are present in a wide variety of systems ranging from small organic and inorganic molecules, organometallics, polymers, proteins, virus particles and nucleic acids.^1–3^ Even though an isolated non-covalent interaction is relatively weak compared to that of a covalent bond, the cumulative energies of multiple interactions can be huge thereby controlling numerous structural and functional properties of molecules in their solid as well as solution states.^4,5^ For example, the unusual properties of water, the double helical structure of DNA and protein folding are primarily driven by non-covalent interactions.^6,7^ In addition to the traditional and long studied hydrogen bonding, van der Waals interactions, London dispersion forces, charge transfer complexation, and electrostatic interactions, several other interactions such as halogen bonding have recently been investigated.^8^ The ability of these weak interactions to generate well-ordered superstructures at multiple length scales has been instrumental in molecular recognition, protein–ligand interaction, host–guest chemistry, catalysis and energy storage materials.^9,10^ They have also proved to be an integral part in designing self-assembled soft materials.^11^ Among soft materials, self-assembled supramolecular organo- and hydrogels derived from low molecular weight organic compounds have been growing rapidly over the past 25 years, due to their unique ability to encapsulate and immobilize solvents at extremely low concentrations. The hydrogels as well as organogels have shown potential applications in tissue engineering, biomedicine, regenerative medicine, optoelectronics, sensors, catalysis and material science.^12–19^ Studies towards the rational design of low molecular weight gelators as well as their structural understanding are growing continuously.^20^ It has been hypothesized and experimentally demonstrated that gel formation is due to self-assembled fibrillar networks (SAFINs) of gelators and the hypothesis is accepted by the scientific community in general.^15^ However, there remained some ambiguities, which are either partially answered or remained challenging to address. Evidence for the presence of fibrillar network structures has been provided using cryo-transmission electron microscopy (TEM), environmental scanning electron microscopy (ESEM) and atomic force microscopy (AFM) studies.^13,15,16^ At the same time, direct information about packing in the native gel and the molecular interactions involved in gelation remained a challenge.

A systematic analysis of the existence and evaluation of the nature of molecular and supramolecular interactions in the solid state is often approached using single crystal and powder X-ray powder diffraction, small-angle X-ray scattering (SAXS) or small angle neutron scattering (SANS) analysis.^13^ However, in a number of cases the compounds under investigation fail to form suitable crystals for X-ray single crystal diffraction or the environment under study is unsuitable for X-ray diffraction. Moreover, the above requirements also restrict the studies of amorphous materials, powder samples, polymers and soft materials. Nuclear magnetic resonance (NMR) spectroscopy, on the other hand, has played a significant role in the analysis and structural characterization of small organic and inorganic molecules, hybrids, macrostructures, biomolecules and single cells.^21^ Notably, the initial attempts have been to develop and apply the methods in solution state experiments.^22^ However, the possibility of using nuclear absorption for solid crystals has been realized soon after its invention.^23^ Since then NMR spectroscopy has continued to evolve as an inevitable part of chemical analysis, structural chemistry and molecular dynamics studies of small organic molecules, polymers, and biomolecules as well as inorganic materials. Coupled with rapid progress in the field of NMR spectroscopy, solid state (SS) cross polarization (CP) and high resolution (HR) magic angle spinning (MAS) NMR spectroscopy is becoming an integral part of structural chemistry, material science and biology.^24–26^ Solid state NMR spectroscopy provides a unique opportunity to study non-crystalline materials, amorphous powders, liquid crystals, gels and even living cells.^27^ Owing to its ability to distinguish the number of different polymorphs, conformational isomers and non-equivalent molecules present in an asymmetric unit of a crystal lattice, this technique has emerged as a complementary tool to X-ray diffraction.^28^ Furthermore, solid state NMR is a non-destructive and non-invasive method, and as a result the samples can be recovered completely and used for further analysis.

Historically, in 1948 Pake in his classic experiment observed a fine structure in the nuclear magnetic resonance absorption for protons, while studying crystalline gypsum (CaSO_4_·2H_2_O).^29^ This experiment proved that NMR spectra for solids can provide useful structural information and is a potential complementary tool to X-ray diffraction. In another experiment, Andrew *et al.* solved the ambiguity regarding the position of hydrogens in urea using solid state NMR.^30^ Since then a number of inorganic salts and organic crystals have been attempted. The historical development of SS NMR is beyond the scope of this review and has been reviewed elsewhere.^23–25^

Over the past six decades solid state NMR has progressed continuously and is becoming a routine tool in structural chemistry and biology.^26^ This is attributed to the seminal paper by Schaefer and Stejskal in 1976, which combined magic angle (54.74°) spinning (MAS), cross polarization (CP) from proton-to-carbon and high power proton decoupling commonly known as CP MAS NMR.^31^

The above developments have led to a new field in solid state science, known as NMR crystallography. Though there was some hesitation to accept the term “*NMR crystallography*” by the crystallographic community, it was for the first time clarified by Elena *et al.* in their paper,^32^ and a reference footnote stating, *“Interestingly, Crystallography is often assimilated today to X-ray studies on single crystals, due to the phenomenal success of this method. Crystallography is obviously a much wider discipline, defined (according to the Encyclopedia Britannica) as “the branch of science that deals with discerning the arrangement and bonding of atoms in crystalline solids and with the geometric structure of crystal lattices.” Since the powders we study here are microcrystalline, the term NMR Crystallography appears natural*.*”*

**Fig. 1:**
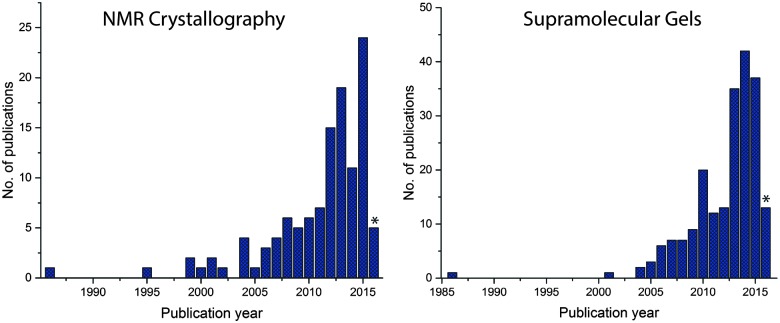
The number of publications *vs.* year of publications using keywords “NMR crystallography” and “supramolecular gels” as entered in SciFinder as of 21.04.2016. Note: keywords such as molecular gels, organogels and hydrogels lead to 115, 1705, 71952 hits respectively. * The numbers for 2016 are incomplete.

Since 2000, there has been rapid progress within the field of NMR crystallography (Fig. 1).^33,34^ In this review, we briefly mention representative examples from the literature, especially how SS NMR coupled with other techniques has been used to study the self-assembled low molecular weight gels and gelators. Furthermore, we discuss how to derive the packing patterns of low molecular weight gelators in their solid and native gel states.

**Fig. 2:**
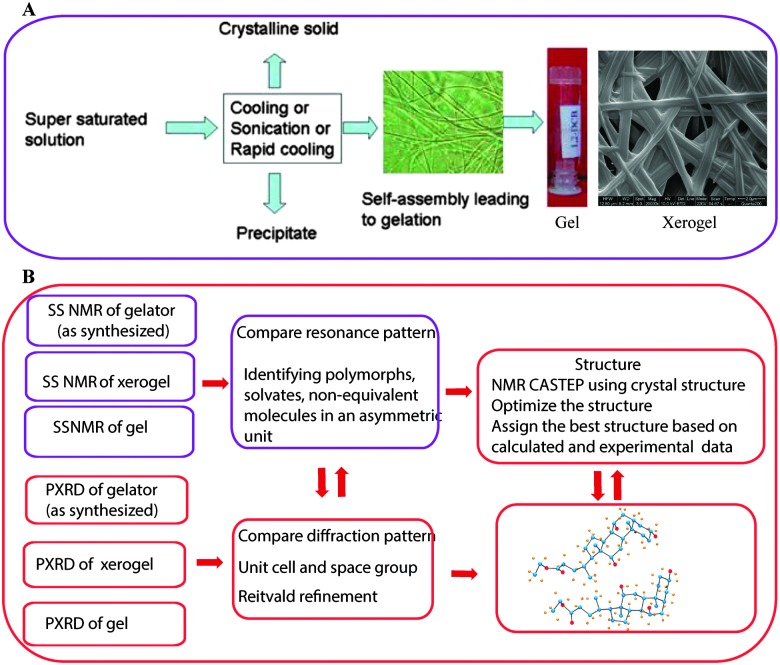
(A) The gelation mechanism of low molecular weight gelators. Reproduced with permission from ref. 43 copyright © 2010 Royal Society of Chemistry; (B) various steps involved in NMR crystallography of gels and gelators.

NMR crystallography of gels or gelators utilizes a combination of techniques to gain complete structural details. One or more approaches have been followed and can be summarized as shown in Fig. 2. It is important to note that “NMR crystallography” based on various solid state NMR techniques combined with XRD is aimed for the study of crystalline structures. However, SS NMR is also useful for the characterization of non-crystalline amorphous materials including liquid crystals, supramolecular gels and other soft materials where XRD has limited resolution. In the case of supramolecular gels SS NMR combined with various microscopic (SEM, TEM, AFM, optical) techniques can provide a useful correlation between gels, xerogels and precipitated solid materials. Importantly, GIPAW calculation methods useful for crystalline structures are not suitable in the case of gels as they lack periodicity in their structures. However, combined solid-state NMR with first principles calculations has shown that due to high sensitivity, SS NMR provides certain advantages to study systems that are disordered or lack periodicity as well as materials where some dynamics are present.^33^ More importantly, it has been shown that the presence of monomorphic and polymorphic gel networks can be detected in a hydrogel matrix.^34^

### Solid state NMR of gels

**Fig. 3:**
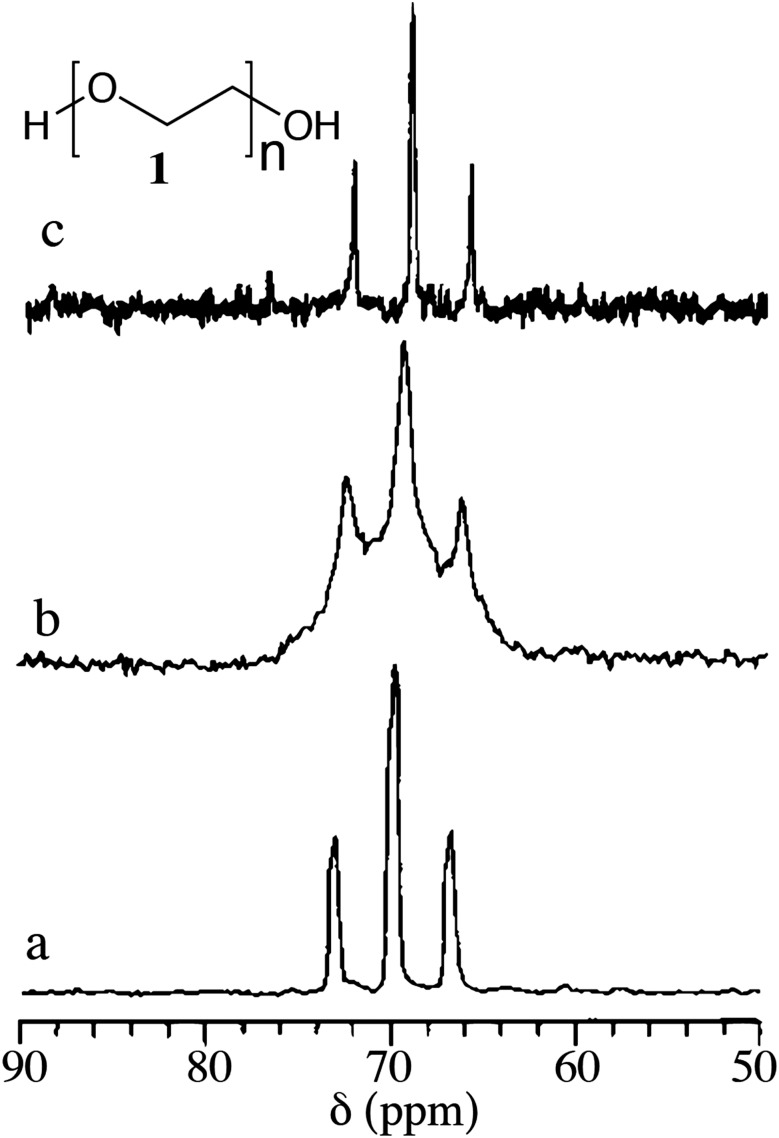
^13^C MAS solid state NMR spectra of polyethylene oxide (PEO, **1**). (a) ^13^C MAS NMR spectrum of gel containing 70% PEO and 30% of water at 4 kHz; (b) ^13^C MAS NMR spectrum of gel containing 70% PEO and 30% of water in a static rotor and (c) ^13^C NMR spectrum of 5% PEO solution in the liquid state probe. Reproduced with permission from ref. 36 Copyright © 1989 Elsevier Inc.

Gels (polymeric and low molecular mass) are complex viscoelastic fluids either physically or chemically cross-linked with highly entangled networks. The increased viscosity of the medium restricts tumbling thereby reducing the molecular motion. This also affects chemical shift anisotropy, and dipolar and quadrupolar interactions. As a result, the solution state NMR spectra of gels/viscous liquids display broad signals. Therefore, it is a challenge to extract the information related to chemical and structural details. However, in 1985 Ford *et al.* in their experiment used polystyrene swollen in CDCl_3_ and demonstrated that solid state MAS NMR spectra can be obtained at 4 kHz spinning frequency.^35^ Ginter *et al.* reported the possibility of using MAS NMR for polyethylene oxide (PEO, MW 3800) hydrogel (Fig. 3).^36^

They compared the ^13^C NMR spectra of 5 wt% PEO solution in water in its solution state with that of 70 wt% PEO gel in water by placing the gel in a 7 mm (od) zirconia rotor. The ^13^C NMR spectra in the solid state under static conditions and magic angle spinning at 4 kHz and the resonance patterns of methylene carbon at 69 ppm were compared. The solid state ^13^C MAS NMR spectra displayed a feature similar to the solution NMR spectra but with broad and overlapping signals, suggesting that there are more than one co-existing phases in the gel state. It was attributed to the presence of two different phases *viz.* mobile and immobile components present in the gel.

**Fig. 4:**
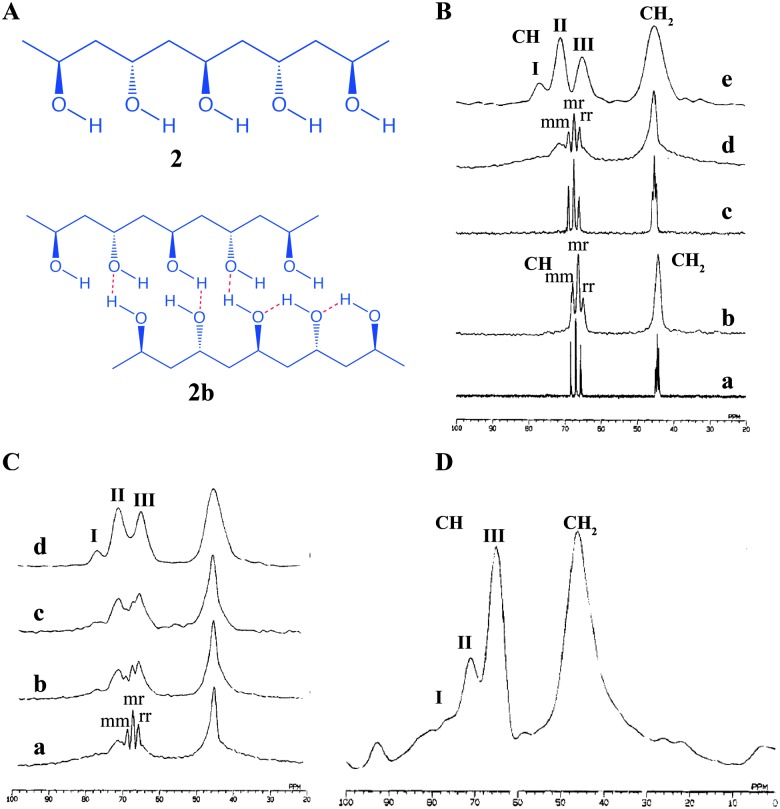
Chemical structure of (A) polyvinyl alcohol (PVA, **2a**) and various hydrogen bonding interactions (**2b**); (B) (a) solution state ^13^C NMR spectrum of PVA/D_2_O solution; (b) solution state ^13^C NMR spectrum in the gel state; (c) solid state ^13^C PST MAS NMR spectrum of the gel; (d) solid state ^13^C CP MAS NMR spectrum of the gel (e) ^13^C CP MAS NMR spectrum of the solid; (C) 67.8 MHz ^13^C CP MAS NMR spectrum of PVA in the gel state: (a) 9.1% PVA gel prepared by freeze–thaw cycles; (b–d) 11.8, 13.8 and 35.0% PVA gels prepared by vaporizing water from the gel and (D) 67.8 MHz ^13^C CP MAS NMR spectrum of PVA gel at –50 °C. Reproduced with permission from ref. 37 Copyright © 1995 American Chemical Society.

Similarly, Kobayashi *et al.* reported an extended study on polyvinyl alcohol (PVA, **2**) gel and extracted the structural details of mobile and immobile components present in the gel (Fig. 4).^37^ The chemical shift values and splitting patterns of ^13^C signals arising from –CH_2_ and –CH in the solution state, gel state and solid state were compared. The solution of PVA in D_2_O showed the splitting of –CH and –CH_2_ into multiplets, which is attributed to their stereochemical configuration. The –CH peaks which are split into triads are assigned as *mm*, *mr* and *rr* (*m* = *meso*, *r* = *racemic*). The –CH_2_ signal splitting arises from the tetrad configuration. Interestingly, in the ^13^C NMR spectrum of gel, –CH shows a triplet and –CH_2_ showed a broad peak (Fig. 4B(b)). The ^13^C pulse-saturation transfer (PST) MAS NMR of PVA gels showed similar features as that of the solution state NMR of PVA gel signals (Fig. 4B(c)). It was suggested that the above observation was due to the fact that only mobile components are visible in the gels under PST MAS conditions. The solid state ^13^C CP MAS NMR spectrum of solid PVA, displayed –CH carbon with three splitting peaks. Notably, the chemical shift difference between these peaks was considerably larger than the splitting due to stereochemical configuration. This is due to splitting by the number of intramolecular hydrogen bonds between the neighbouring hydroxyl groups (Fig. 2B and 4A). The peaks show the chemical shifts depending on the number of hydrogen bonds involved. Important observations were made by recording the ^13^C CP MAS NMR of PVA gel, wherein it was found that there are splitting patterns, which resemble the ^13^C NMR of PVA gel as well as that of the ^13^C CP MAS NMR of the solid PVA. Therefore, ^13^C CP MAS has the ability to detect both mobile and immobile components. It was further shown that upon increasing the concentration of PVA the ^13^C CP MAS NMR tends to behave more like that of a solid as the stereochemical configurations are decreased. PVA gel with a concentration of 35%, behaved similar to solid in its spectral features. It is important to note that ^13^C CP MAS NMR relies on the principle of magnetization transfer from protons to carbons *via* a dipolar coupling mechanism. This process is highly effective in immobile components such as solids but the efficiency is greatly reduced for mobile components. In such situations a combination of techniques which do not rely on magnetization transfer instead utilizes direct single pulse ^13^C MAS NMR is useful as they provide information related to mobile components.

An extensive review on solid state NMR of polymer gels is beyond the scope of this review. However, the above studies form the basis for the investigation of many supramolecular gels. In particular, the solution state NMR spectroscopy of low molecular weight gels has been discussed based on the concept of mobile and immobile components as well as the components which are bound to the gel network and free molecules.^38^ Recently, DOSY NMR experiments and HRMAS studies have been shown to support this hypothesis.^39^ The details of such studies have been reviewed elsewhere.^40^ Therefore, the following part of the review we will focus towards the solid state NMR of low molecular weight gelators with a special emphasis on CP MAS NMR techniques.

**Fig. 5:**
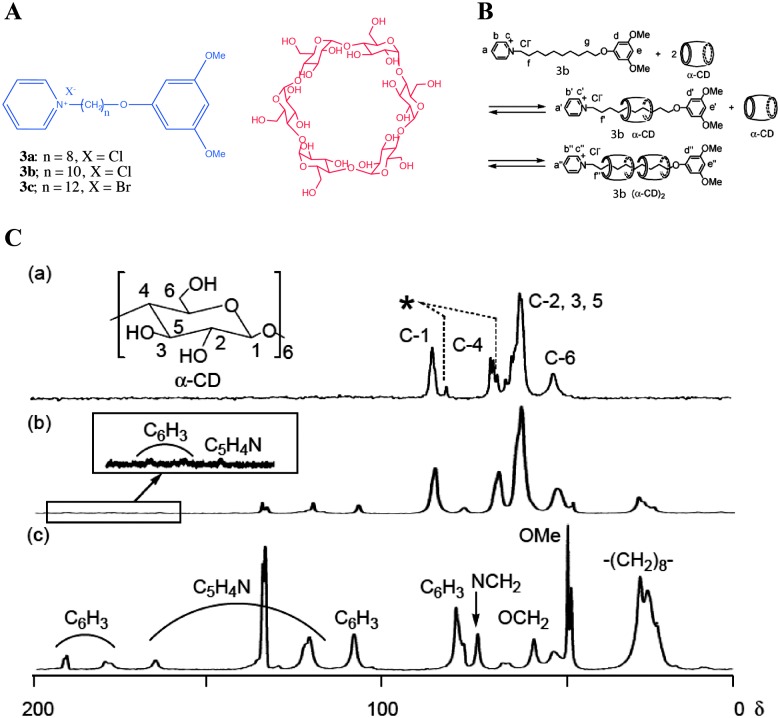
Pseudorotaxane based gelators: (A) Chemical structures of alkylpyridinium halides and α-cyclodextrin, (B) pseudorotaxane formation leading to gelation: (C) ^13^C CP MAS NMR spectra of (a) α-CD; (b) xerogel of **3b** and α-CD, and (c) **3b**. Peaks with an asterisk are assigned to C-1 and C-4 with a conformationally strained glycoside linkage. Reproduced with permission from ref. 41 Copyright © 2009 Royal Society of Chemistry.

Unlike polymeric gels, the low molecular weight gels are composed of supramolecularly self-assembled fibrillar networks of small molecules (*M*_w_ < 3 kDa, Fig. 2A). Importantly, a number of gels are studied based on their xerogels or aerogels, and packing patterns are deciphered based on single crystal structures.^40^ However, most of the gelators do not undergo crystallization. Therefore, the packing pattern leading to the process of gelation is still poorly understood and there is a debate on the presence of 3D networks in the native gel and its similarity/identity to those observed in the xerogels. This question arises because upon sample preparation, during evaporation of the solvent or under experimental conditions (high vacuum in SEM, TEM), there may be a change in the structure and as a result the morphology in the gel state may differ significantly from that of the xerogel. There exist a few reports in the literature, in which suggestions have been made regarding the mode of the packing pattern and a correlation between the bulk solid and xerogels mostly evidenced by powder X-ray diffraction (PXRD), small angle neutron scattering (SANS), and small angle X-ray scattering (SAXS) techniques.^40^ Most of the gelators do not undergo crystallization and therefore, a complete understanding and clear evidence of the gelation process remained undisclosed. However, a few reports which describe a correlation between the single crystal and the interactions which lead to gelation have been reported in the literature. More importantly, gelation conditions (concentration, temperature and solvent) are significantly different from the crystallization conditions. Therefore, packing patterns in the gel or xerogel may differ drastically from the crystal structure. One way to overcome this ambiguity is by combining more than one analytical techniques, which are complementary to each other. In this context, solid state NMR spectroscopy provides useful information when combined with X-ray powder diffraction and other complementary tools. Taira *et al.* reported the gelation of alkylpyrdinium derivatives (Fig. 3a–c and 5) in the presence of α-cyclodextrin (α-CD).^41^ By combining the MALDI-TOF results with ^13^C CP MAS of NMR spectroscopy, the authors concluded that the gelation is due to pseudorotaxane formation.

In more detail, the ^13^C CP MAS NMR spectrum of xerogel derived from a mixture of **3b** : α-CD (1** **:** **2) hydrogel (Fig. 5C) did not show ^13^C NMR signals at 81 and 98 ppm, which are unique to C-1 and C-4 carbons of free α-CD. This suggests that xerogel is composed of pseudorotaxane. Therefore, the original hydrogel is composed of pseudorotaxane and not free CD (Fig. 5B). However, there was no evidence based on solid state NMR of the native gel.

**Fig. 6:**
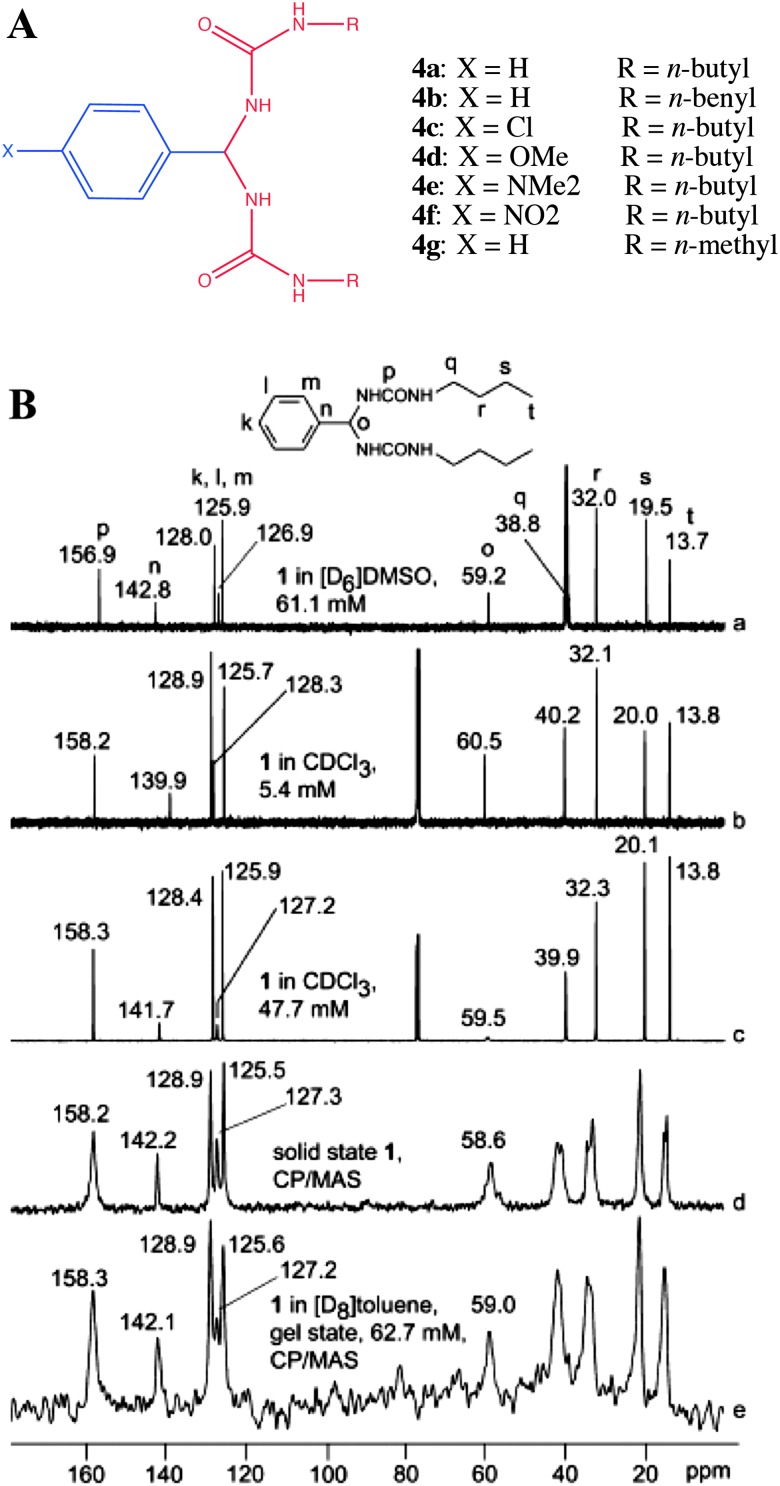
(A) Chemical structures of bis-urea benzene derivatives; (B) comparison of solution, gel and solid state ^13^C NMR spectra of **4a**: (a) 61.1 mM in DMSO-*d*_6_; (b) 5.4 mM in CDCl_3_; (c) 47.7 mM in CDCl_3_; (d) ^13^C CP MAS NMR of the solid and (e) toluene-*d*_8_ gel of **4a**. Reproduced with permission from ref. 42 © Wiley-VCH.

In this direction Schoonbeek *et al.* studied the properties of 1,2-bis-urea benzene derivatives (**4a–g**, Fig. 6) and compared the solution NMR, solid state ^13^C CP MAS NMR of the solid gelator and CP MAS NMR of toluene-*d*_8_ gel (Fig. 6B).^42^ There were no significant changes in the position of ^13^C NMR signals between solution at various concentrations, solid and gel states. It was attributed to the fact that there exists an intramolecular H-bonding within the gelator molecules in their solution state and also strong solvent–solute interactions, which essentially have the same effect as strong inter- and intramolecular interactions in the solid and gel state.

More importantly, both the carbonyls were found to be equivalent in solution, gel and solid states. Furthermore, the phenyl carbons in the solid state showed no splitting; however, the butyl carbons did show splitting in their solid state NMR spectra. Though there is no clear explanation for this observation, this might be a result of a severe disorder in the flexible butyl chains. Even in this work no conclusive evidence was obtained from the gel state due to poor spectral resolution.

**Fig. 7:**
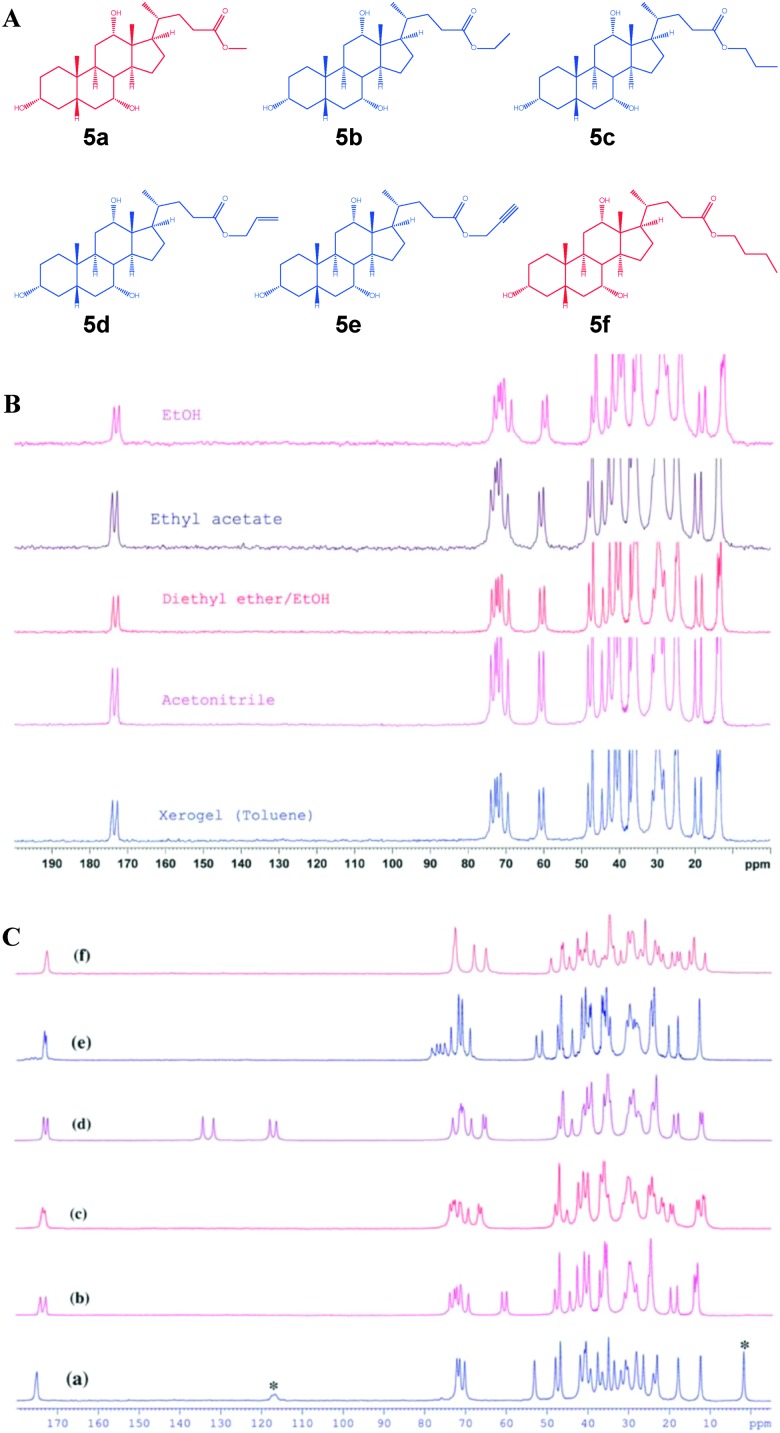
Cholic acid ester based gelators: (A) chemical structures of gelators and non-gelators; (B) solid state^13^C CP MAS NMR of xerogel and recrystallized samples of ethyl cholate **5d** from different solvents. (C) ^13^C CP MAS NMR spectra of **5a–5f**, indicating that all the gelators show a doublet resonance pattern, whereas **5a** forms a solvate and **5f** is relatively less crystalline. (Note: asterisk indicates the signals arising from solvent molecules). Reproduced with permission from ref. 43 Copyright © 2010 Royal Society of Chemistry.

**Fig. 8:**
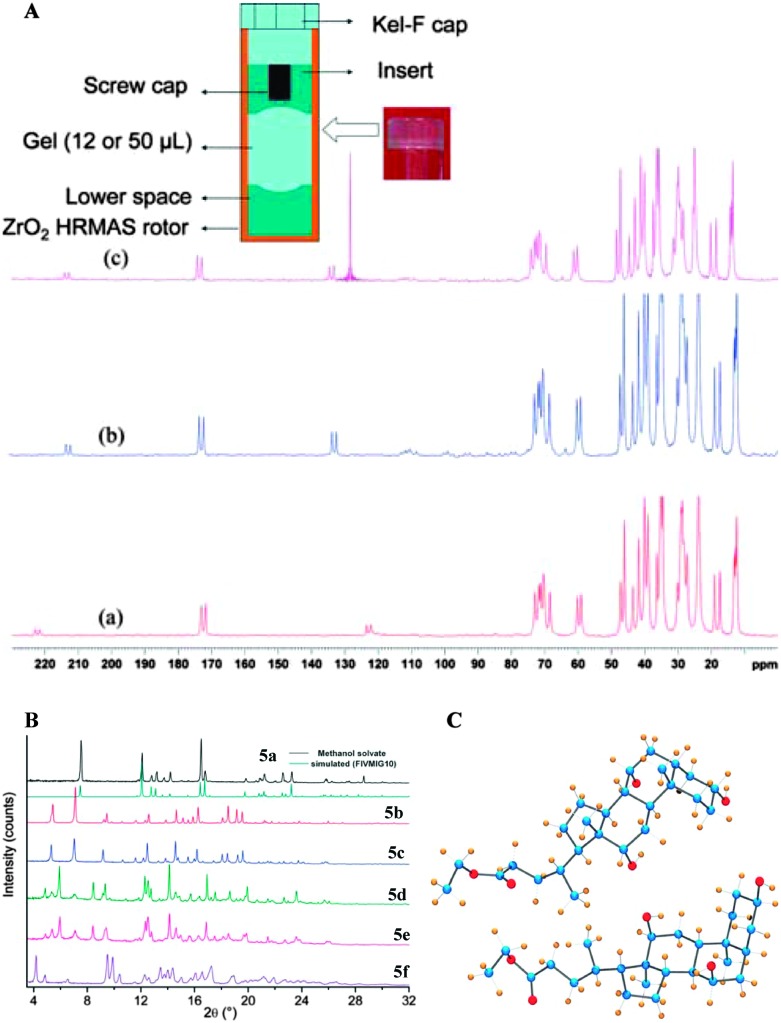
(A) Schematic representation showing the location and preparation of a gel sample for the CP MAS experiment and the ^13^C CP MAS NMR spectra of benzene-*d*_6_ gel of **5b** at (a) 5 kHz and (b) 4 kHz; and (c) benzene gel of **5b**; (B) experimental PXRD patterns of **5a–f**. The similarity of the patterns of **5b** and **5c** indicates isostructural features on their packing modes. A similar trend can be observed between the patterns of **5d** and **5e**; (C) X-ray structure of **5b** showing two molecules in an asymmetric unit. Carbon (blue), oxygen (red) and hydrogen (gold). Reproduced with permission from ref. 43 © Royal Society of Chemistry.

In 2010, Nonappa *et al.* have shown that by combining solid state ^13^C CP MAS NMR, X-ray powder diffraction and thermoanalytical techniques, the packing patterns in the gel and xerogel states can be elucidated.^43^ This study represents one of the first examples of a gel studied in its native gel state (using benzene, previously deuterated solvents were used). This forms the basis for the discussion in the following part of the manuscript. Using simple esters of cholic acid, a number of gelators were prepared (Fig. 7).^44^ The gel formation was observed only for ethyl **5b**, propyl **5c**, allyl **5d** and propargyl **5e** esters of cholic acid. The methyl derivative **5a** readily formed crystals, whereas the butyl derivative **5f** remained in the solution in the tested solvents. Based on SEM, AFM and polarizing optical microscopy images of the xerogles it was found that the gelators undergo self-assembly into 300 nm (diameter) fibers with indefinite length. Furthermore, the direct evolution of fibers was supported using optical microscopy imaging.^43^ In order to gain the interactions involved in gel formation and packing patterns the solution state NMR of the gel, and the solid state ^13^C CP MAS NMR of the xerogels and native gels were combined with X-ray powder diffraction. The ^13^C CP MAS NMR of the synthetic solid and the xerogel displayed a doublet resonance pattern (Fig. 7B), which was a consequence of two non-equivalent molecules in the asymmetric unit. Furthermore, using very careful sample preparation the solid state ^13^C CP MAS NMR spectra of gels were compared. Interestingly, the gels when spun at 4, 5 and 8 kHz showed a similar resonance pattern as that of the xerogel (Fig. 8A).

The above observation suggested that the packing pattern in the gel state and the xerogel is similar. Using X-ray powder diffraction indexing and Rietveld refinement, the solid state structure of the gelator was solved (Fig. 8B and C). This represents the first example of a gelator crystal structure solved by combining the solid state NMR and X-ray powder diffraction analysis. As revealed by ^13^C CP MAS studies, the crystal structure displayed two non-equivalent molecules in an asymmetric unit of a crystal lattice. More importantly, the non gelators either formed solvates or amorphous solids. Interestingly, all the gelators followed similar packing patterns, whereas the non-gelators showed a singlet resonance pattern. This suggests that the packing patterns are important in determining whether a given molecule undergoes crystallization into single crystals, solvates, amorphous solids or gels.

**Fig. 9:**
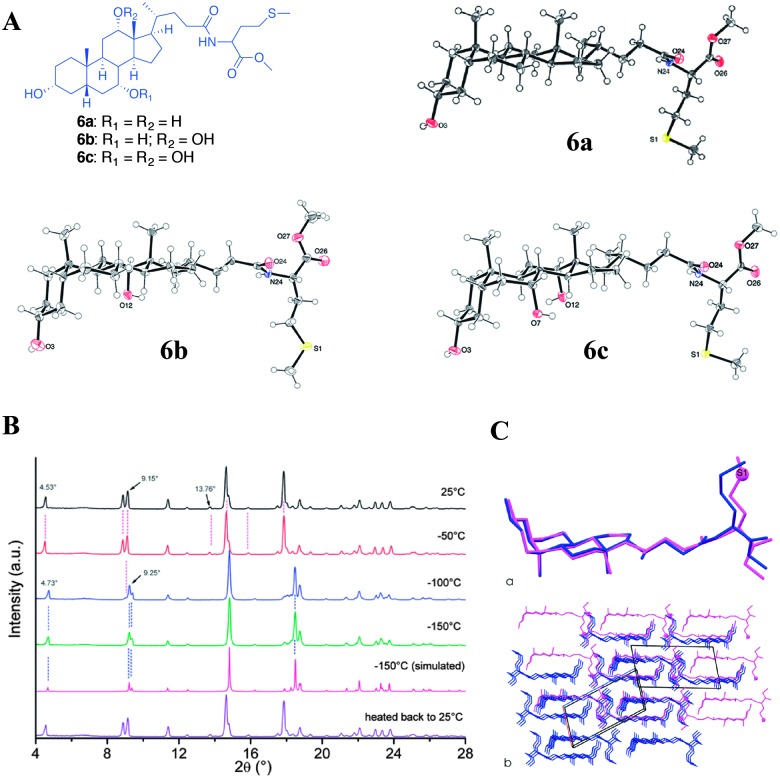
(A) Chemical structures of bile acid-l-methionine methyl ester conjugates and ORTEP10 plots of asymmetric units of compounds **6a–c**; (B) temperature-dependent diffraction patterns of xerogel of compound **6a** together with a simulated pattern of the low-temperature single crystal structure **6a**; (C) overlays of (a) molecular structures and (b) packing modes of single crystal X-ray structure (dark structure) and structure solved using XRPD (light structure) presented along the *b*-axis of the room-temperature structure **6a**. For clarity, the sulfur atom (s1) of room temperature structure is presented in a ball-style and hydrogen atoms are omitted. Reproduced with permission from ref. 45 © Royal Society of Chemistry.

Noponen *et al.* reported the organogelation of bile acid-l-methionine methyl ester conjugates and compared the solid state single crystal X-ray structure, ^13^C CP MAS NMR and X-ray powder diffraction patterns (Fig. 9).^45^ Compounds **6a–c** underwent crystallization leading to quality single crystals suitable for structural determination. However, only **6b** and **6c** showed the ability to form gels in aromatic solvents.

**Fig. 10:**
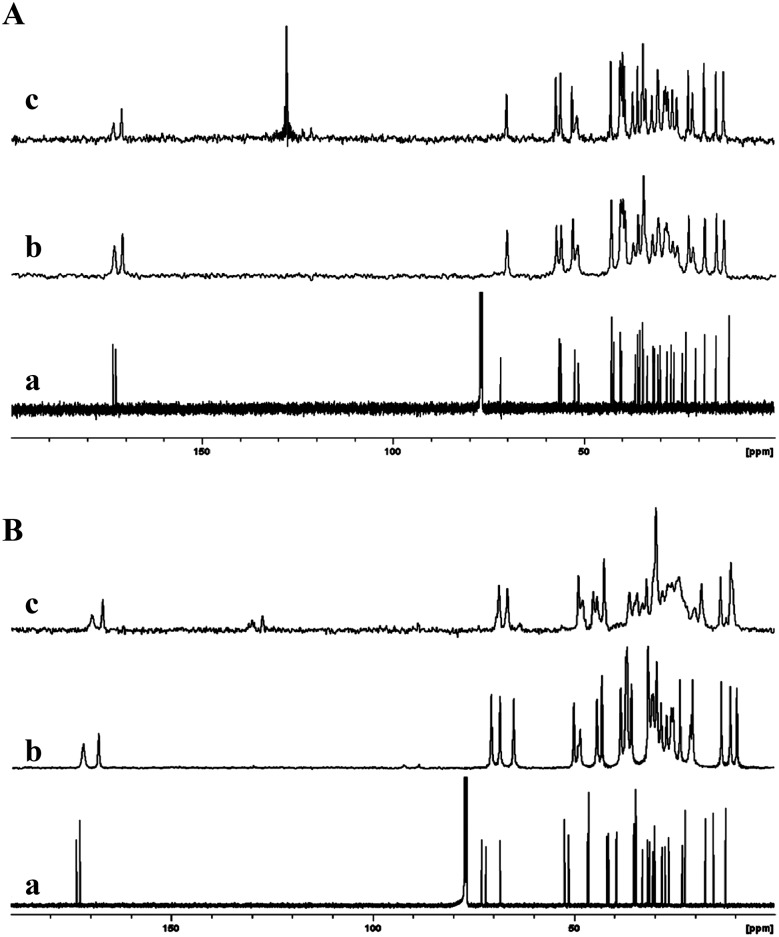
(A) ^13^C NMR spectra of compound **6b**; (a) solution state; (b) ^13^CP MAS NMR spectrum of the synthesis product recrystallized from acetonitrile; (c) ^13^C CP MAS NMR spectrum of 2% (w/v) benzene gel. (B) ^13^C NMR spectra of compound **6c**; (a) solution state; (b) ^13^C CP MAS NMR spectrum of the synthesis product recrystallized from acetonitrile and (c) ^13^C CP MAS NMR spectrum of 4% (w/v) toluene-*d*_8_ gel. Reproduced with permission from ref. 45 Copyright © 2010 Royal Society of Chemistry.

The crystallization studies were carried out in acetonitrile and the solid state ^13^C CP MAS NMR spectra of recrystallized samples were compared with the benzene-*d*_6_ and toluene-*d*_8_ gels. It was found that compound **6a** shows a similar spectral pattern for recrystallized sample in acetonitrile as well as native gel, suggesting that the gelator molecules have a similar packing pattern in their gel state and in single crystals. This was further supported using the X-ray powder diffraction patterns of xerogels. The experimental diffraction patterns of xerogels were compared with that of the simulated powder diffraction patterns from the single crystal X-ray structure. On the other hand, compound **6b** showed a different spectral pattern for the single crystals and native gel (Fig. 10). This suggests that the packing mode in the gel state significantly differed from that of the single crystal structure for **6b**.

**Fig. 11:**
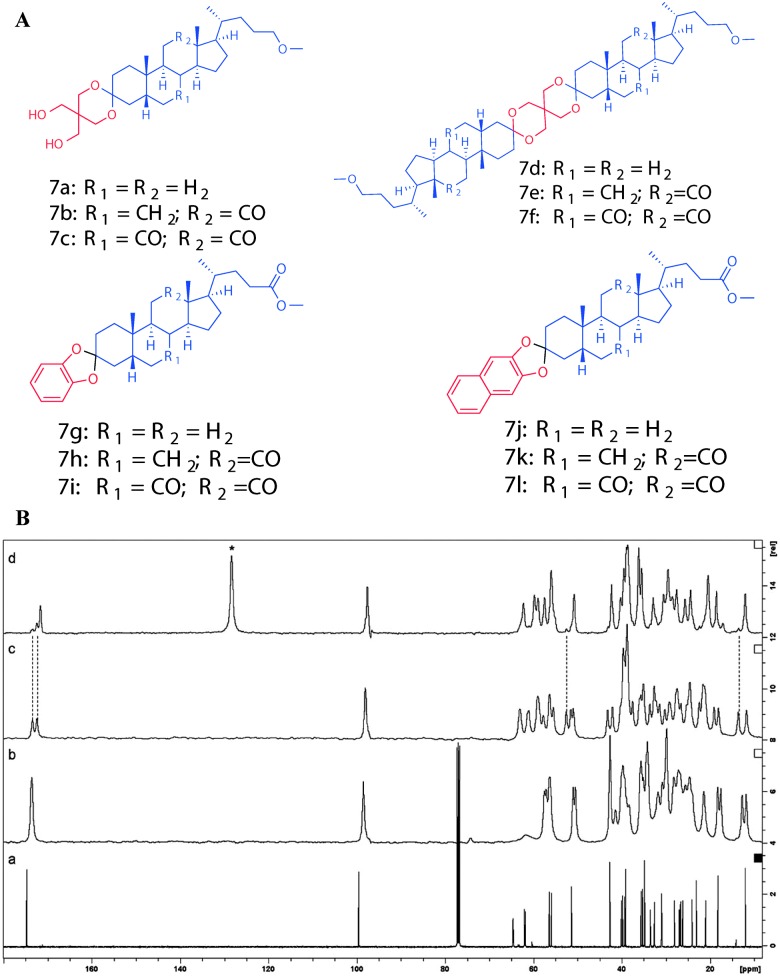
(A) Chemical structures of mono- and diketal derivatives of bile acids. (B) ^13^C NMR spectrum of **7a** in CDCl_3_ (a) and ^13^C CP MAS NMR spectra of **7a** crystallized from acetonitrile (b), toluene (c), and benzene (d). Reproduced with permission from ref. 46 Copyright © 2010 Royal Society of Chemistry.

In another study, Ikonen *et al.* reported the mono- and diketal derivatives of bile acids using pentaerythritol, catechol and 2,3-dihydroxy naphthalene.^46^ The monoketal derivatives of pentaerythritol displayed gelation ability in organic solvents (Fig. 11A), while the diketals from pentaerythritol as well as the ketals from catechol and 2,3-dihydroxy naphthalene formed single crystals. Gelator **7a** was recrystallized from various solvents (benzene, toluene, *p*-xylene, chlorobenzene, acetonitrile and acetone) and studied using ^13^C CP MAS NMR spectroscopy. The solid state ^13^C CP MAS NMR spectra were compared with respective solution state ^13^C NMR spectral data. A systematic analysis of ^13^C CP MAS NMR spectra of compound **7a** recrystallized from toluene, *p*-xylene, and chlorobenzene revealed a doublet resonance pattern indicating the presence of two crystallographically independent molecules per asymmetric unit (Fig. 11B(c)). Interestingly, the sample crystallized from benzene, was found to contain two different polymorphic forms. The minor polymorphic form was found to display similar spectral patterns as the ones crystallized from the other aromatic solvents (Fig. 11B(d)), whereas the major form was a benzene solvate. Furthermore, recrystallization of **7a** from non-gelling solvents such as acetonitrile or acetone displayed a doublet resonance pattern. However, the doublet resonance patterns were not observed for all the carbons and the signals from pentaerythritol carbons were not well resolved, presumably due to a severe disorder in that part of the gelator molecule. More importantly, recrystallization of gelator **7a** from gel-forming solvents displayed well resolved signals in their ^13^C CP MAS NMR spectra suggesting a highly ordered packing system in the gel state.

**Fig. 12:**
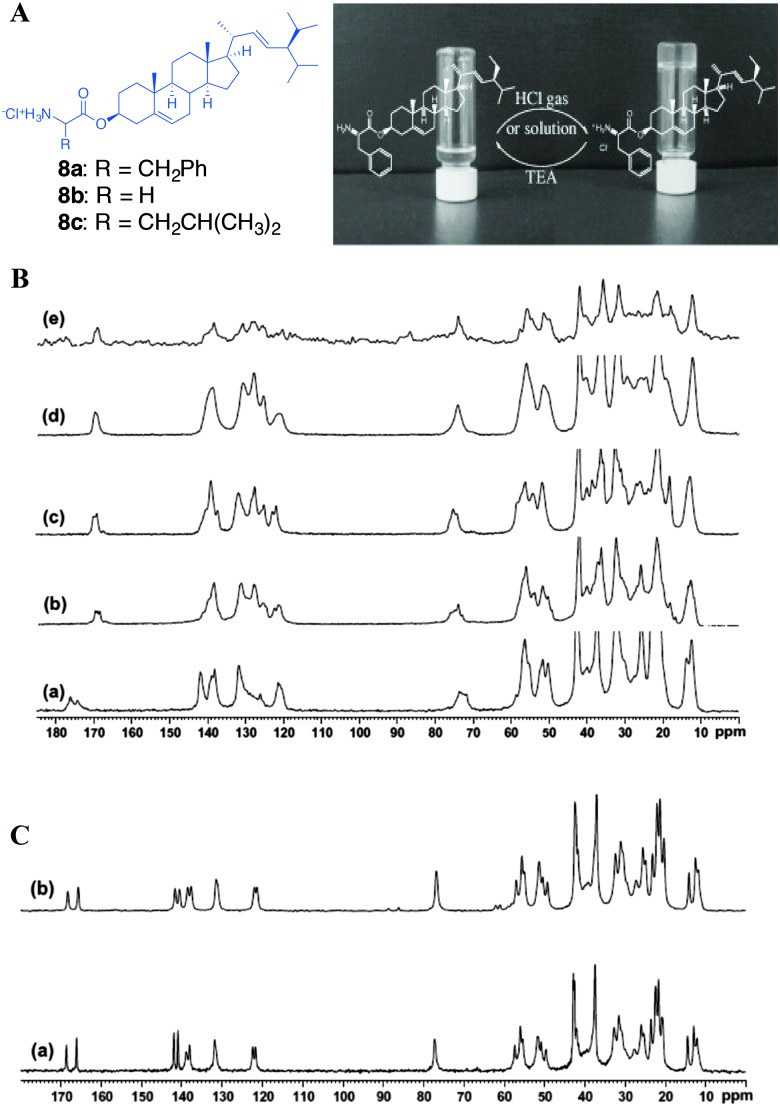
(A) Chemical structures of stigmasterol–amino acid conjugates and protonation and deprotonation of stigmasteryl phenylalaninate in tetrachloromethane; (B) ^13^C CP MAS NMR of (a) **8a** solid; (b) **8b** solid, and xerogels of **8b** obtained from (c) benzene gel of **8a**; (d) from CCl_4_ gel of **8a**, and (e) CCl_4_ gel of **8b** (4% w/v). (C) ^13^C solid state NMR of (a) **8b** solid and (b) xerogel from 1-butanol gel of **8b**. Reproduced with permission from ref. 47, Copyright © 2011 Elsevier Inc.

Svobodová *et al.* reported the stigmasterol-aminoacid (glycine, l-leucine and l-phenyl alanine) conjugates as stimuli responsive gelators (Fig. 12).^47^ The hydrochloride salts of the conjugates underwent self-assembly leading to organogelation upon dissolving in a number of alcoholic and aromatic solvents. The ^13^C CP MAS NMR spectra were recorded for the synthetic solids and the spectral patterns were compared with that of the gels as well xerogels. The structural studies were performed for **8a** and **8b**. The ^13^C CP MAS NMR of the synthetic solid showed to be crystalline in nature but the signals were relatively broad. However, a careful analysis of the^13^C CP MAS NMR spectral data of **8a** revealed three signals for carbonyl carbon at 169.88, 169.34 and 168.76 ppm suggesting the presence of more than one polymorphic forms (Fig. 12B). This was further confirmed by performing the ^15^N CP MAS NMR experiment, which indicated the presence of more than one ^15^N signals. Interestingly, the ^13^C CP MAS NMR of the xerogel prepared from the benzene gel of **8a** showed better crystallinity compared to that of the synthetic solid (Fig. 12B(e)). More importantly a doublet resonance pattern with carbonyl signals at 169.85 and 168.92 ppm was observed. This suggests that there are two crystallographically independent molecules in an asymmetric unit.

In addition to the above studies, the authors studied the xerogel obtained from the CCl_4_-gel, which displayed a singlet resonance pattern. This observation revealed that the synthetic solid was a mixture of polymorphs and can be separated by changing the solvents. Furthermore, the comparison of ^13^C CP MAS NMR of CCl_4_ native gel with that of the xerogel confirmed that both the gel and xerogels have similar packing patterns. The above observation was further supported using the morphological features of xerogels obtained using SEM, which suggest that they are highly solvent dependent. On the other hand, the ^13^C CP MAS NMR spectrum of **8b** displayed a doublet resonance pattern. The comparison of ^13^C CP MAS NMR and ^15^N CP MAS NMR of the 1-butanol gel of **8b** with that of xerogel confirmed the similarity between synthetic solid, xerogel and the gel packing patterns.

**Fig. 13:**
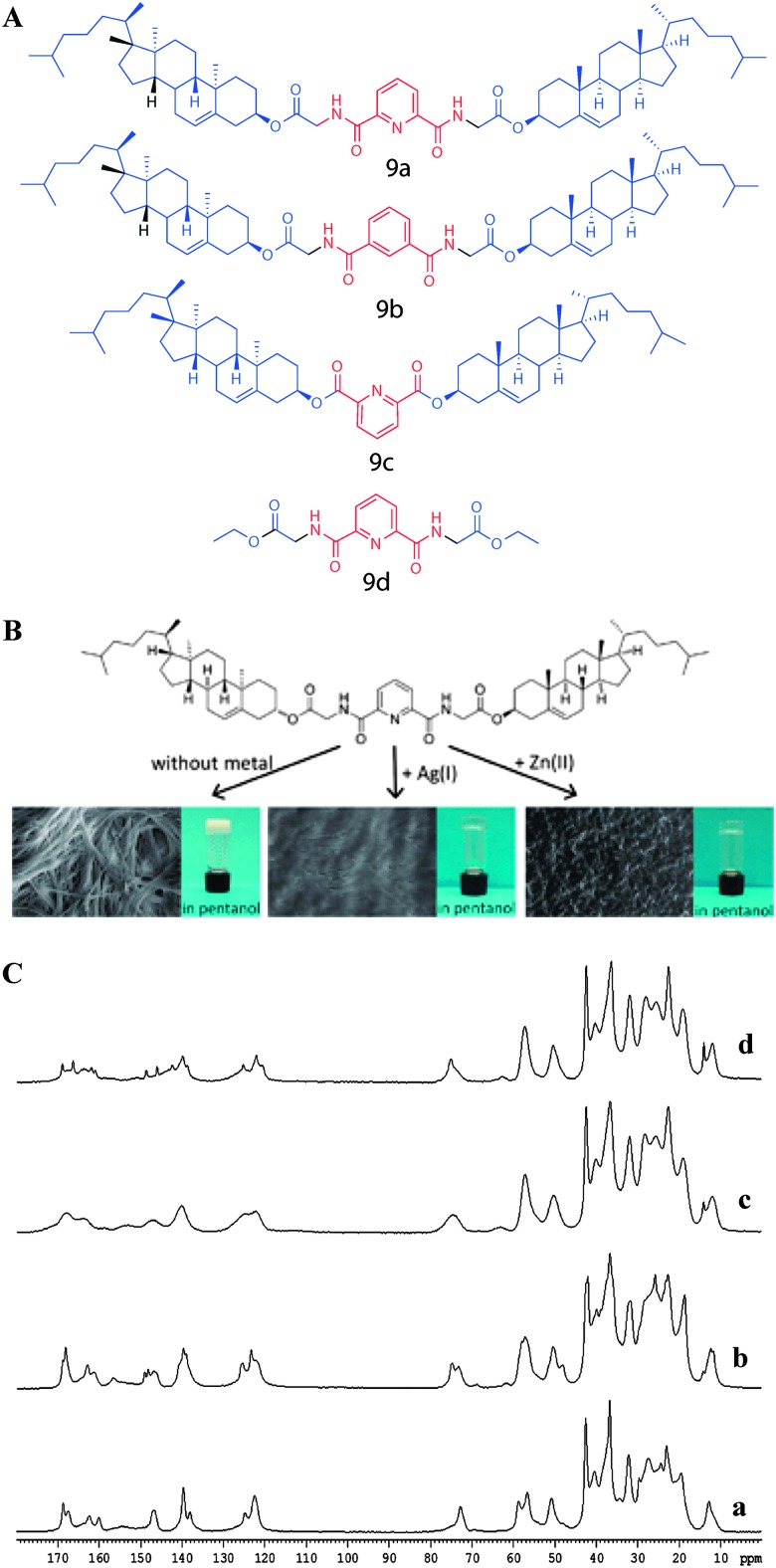
(A) Chemical structures of metallosupramolecular gelators. (B) Structure of compound **9a**, a gelator of the A(LS)_2_ type, with marked non-covalent interaction sites. (C) ^13^C CP MAS NMR spectra of (a) solid of **9a** from CHCl_3_; (b) xerogel of **9a** from pentan-1-ol; (c) xerogel of **9a** + Ag(i) from pentan-1-ol and (d) xerogel of **9a** + Zn(ii) from pentan-1-ol. Reproduced with permission from ref. 48, Copyright © 2012 Royal Society of Chemistry.

In another study, Svobodová *et al.* reported the gelation properties of aromatic-linker-steroid A(LS)_2_ type gelators containing pyridine-2,6-dicarboxylic acid as a linker and cholersterol glycinate as the steroidal moiety (Fig. 13).^48–50^ The molecules also underwent metal complexation induced gelation, leading to metallosupramolecular gels. The molecular packing of gelator **9a** and its metal complexes was performed using combined ^13^C CP MAS NMR and X-ray diffraction experiments. In this study ^13^C CP MAS NMR spectral patterns of compound **9a** crystallized from chloroform and the xerogels from pentan-1-ol gel were compared (Fig. 13). ^13^C CP MAS NMR spectra of the synthetic solid **9a** (Fig. 13C(a)) and its xerogel from pentan-1-ol (Fig. 13C(b)) gel displayed similarities in their resonance pattern suggesting similar molecular packing. The broad signals in the spectrum of the xerogel of **9a** + Ag(i) from pentan-1-ol (Fig. 13C(c)) due to its amorphous nature and was supported by X-ray powder diffraction analysis. The ^13^C CP MAS NMR spectrum of the xerogel of **9a** + Zn(ii) complex from pentan-1-ol gel (Fig. 13C(d)) displayed crystalline nature. However, the spectral patterns deviated from the other xerogels indicating a different packing mode.

**Fig. 14:**
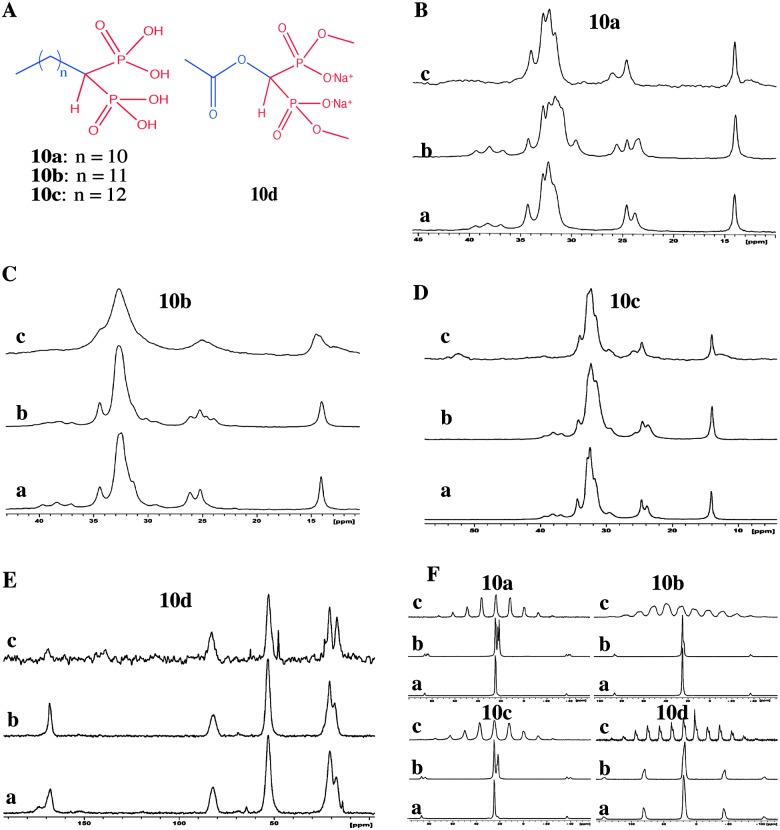
Bisphosphonate hydrogelators: (A) chemical structures of hydrogelators; (B–E) ^13^C CP MAS NMR spectra of (a) synthetic solid; (b) xerogel and (c) gel (4 or 5 w/v%) of **10a–10d**; (F) ^31^P CP MAS NMR spectra of (a) synthetic solid; (b) xerogel and (c) the hydrogel of **10a–10d**. Reproduced with permission from ref. 51, Copyright © 2013 Royal Society of Chemistry.

Alanne *et al.* reported bisphosphonate hydrogelators **10a–10d** (Fig. 14) and used solid state ^13^C CP MAS and ^31^P MAS NMR spectroscopy to compare the solid state structural properties and packing patterns in the gel, xerogel and synthetic solids (Fig. 14A–D).^51^ The authors concluded that the ^13^C CP MAS NMR of the synthetic solids, and the gels resembled each other but differed from that of the xerogels. However, a close examination of the ^13^C CP MAS NMR gives an interesting overview which was not taken into consideration in this work. Careful analysis of the ^13^C NMR spectra of the synthetic product, xerogel and gels shows that they all differ in their spectral pattern. Comparing the solid state NMR spectra of synthetic solid and xerogels suggests that there exists more than one form. Interestingly, the two ^13^C signals appearing in the gel state resemble that of the peaks at around 25 ppm signals (Fig. 14B–D) in their solid state. This suggests that there exists more than one phase in the gel such as mobile and immobile components. Interestingly, the third signal disappeared in the gel state. On other hand, the intensities of ^13^C signals at around 25 ppm are reversed in the gel state compared to that of the xerogel. This suggests that all the three states have different packing patterns. However, the X-ray powder patterns were not supportive for this argument, and it is not a surprise since several examples are known in the literature where powder X-ray diffraction failed to detect a minor polymorphic form in the solid state compared to solid state NMR. In particular, CP MAS NMR is very sensitive in identifying a minute amount of polymorphic forms. On the other hand, similar spectral patterns in ^31^P CP MAS NMR were also shown. For example, for gelator molecule **10a**, there is a significant difference between the synthetic solid and the xerogels. This is in correlation with that of the ^13^C CP MAS NMR studies.

This suggests that a simple correlation between the synthetic solid, xerogel and gels is not always straightforward. In such cases more extended studies such as 2D solid state NMR spectroscopy will be useful for correlation spectroscopy. Therefore, the study of gels and gelators using solid state NMR is still developing and there is more scope to apply this technique and develop into more complex systems.

Polymorphism is an important property of organic compounds and can significantly alter the physical and chemical properties.

**Fig. 15:**
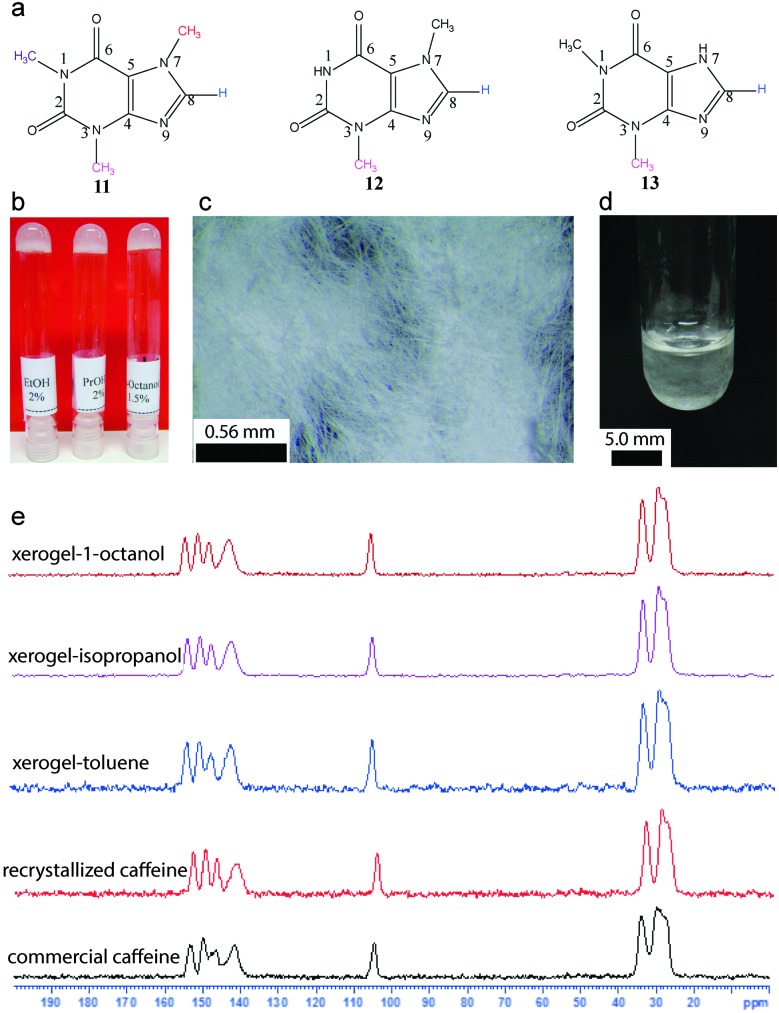
Caffeine as a gelator. (a) Chemical structure of caffeine, theophylline and theobromine; (b) photograph of gels derived from caffeine; (c) optical microscopy image of xerogel; (d) microcrystalline network formation and (e) ^13^C CP MAS NMR of commercial caffeine, recrystallized form and xerogels. Reproduced with permission from ref. 52, Copyright © 2016 by the authors.

Solid state NMR has been extensively utilized to identify and characterize polymorphs.^28^ A given gelator molecule may contain various polymorphs or solvates, which can affect the gelation properties. It has been shown that a recrystallized form of commercial caffeine has the ability to undergo gelation in alcoholic as well as aromatic organic solvents (Fig. 15).

Using ^13^C CP MAS solid state NMR, it was demonstrated that the commercial caffeine contains more than one form. When recrystallized from ethanol, it results in an anhydrous form, which undergoes self-assembly into microcrystalline networks and immobilizes the solvents.^52^

## Conclusions

In conclusion both solid state NMR spectroscopy and supramolecular gels are continuously evolving areas of research with new applications. Solid state NMR coupled with other complementary techniques such as X-ray diffraction, SAXS, SANS, FT-IR, Raman spectroscopy and electron microscopy known as NMR crystallography is becoming an important tool to study molecules, materials and nanostructures in a wide variety of forms. While the pharmaceutical industries concentrate on polymorphs, solvates and salts, the utilization of SS NMR in materials science is growing rapidly. Solid state NMR of gels is not straightforward as there are several disadvantages, especially, due to high spinning speed. This might result in the disintegration of gels due to the centrifugal force generated upon spinning. Therefore, low spinning speeds are generally recommended, which might lead to poor resolution and overlapping spinning side bands. On the other hand, there is a growing interest in developing gels based on new supramolecular interactions such as subcomponent self-assembly and halogen bonding.^8,50^ Solid state NMR is extremely useful for studying such new interactions and multicomponent assemblies.
